# F47. COGNITIVE REMEDIATION AND PHYSICAL EXERCISE IN MULTI-EPISODE SCHIZOPHRENIA: STUDY PROTOCOL FOR A RANDOMISED CONTROLLED TRIAL

**DOI:** 10.1093/schbul/sby017.578

**Published:** 2018-04-01

**Authors:** Nuria Pujol, Víctor Pérez-Solà, Romina Cortizo, Lourdes Ayllon, Teresa Salvador, Daniel Moreno, Ferran Català, Jacobo Chamorro, Silvia Oller, Javier Polo-Velasco, Adelina Abellanas, Cristobal Diez-Aja, Anna Mane

**Affiliations:** 1 Universitat De Barcelona; 2 Institute of Neuropsychiatry and Addiction of the Barcelona MAR Health Park, Spain Hospital del Mar Medical Research Institute, Ciber Salud Mental Group (CIBERSAM); 3 Institute of Neuropsychiatry and Addiction of the Barcelona MAR Health Park

## Abstract

**Background:**

Cognitive remediation (CR) and physical exercise have separately shown promising results in schizophrenia cognitive improvement, despite this, the impact on daily functionality is still limited. Physical exercise increases Brain Derived Neurotrophic Factor (BDNF) levels, promoting neuronal and cognitive plasticity, which can maximize the impact of CR. We are conducting a randomised controlled trial to determine the efficacy of an intensive program that combines CR and physical exercise on cognition and related outcomes for patients with schizophrenia. In addition, we investigate functional and structural brain effects of this intervention and its association to BDNF.

**Methods:**

This study protocol describes a randomized controlled trial in which 74 patients are randomly assigned to either CR and physical exercise or CR and health promotion. The interventions are 12-week long and consist of three weekly sessions (90min of CR and 40min of either aerobic exercise or health promotion). To be included in the study, patients must be diagnosed with schizophrenia or schizoaffective disorder, aged 28–60 years, and do low physical activity, as measured by International Physical Activity Questionnaire, IPAQ. Exclusion Criteria for participation in the study are the presence of neurological or substance use disorders, IQ < 70 and somatic illnesses that contraindicate physical exercise. Healthy control participants (n=18) are screened for the presence of lifetime Axis I psychotic disorders and for the presence of a first-degree relative with schizophrenia. Primary outcome measures are cognitive performance, functional outcome, negative symptoms, BDNF levels and neuroimaging measures. Secondary outcome measures are quality of life and metabolic parameters. All measures are blindly assessed at baseline, at 3 months follow up and at 15 months follow up.

This trial was approved by the Comité Ètic d’Investigació Clínica de l’Hospital del Mar (CEIC) 2015/6209/I

**Results:**

This poster is a study protocol. We will correct data from now on.

**Discussion:**

The results of this trial will provide valuable information about whether cognitive remediation efficacy for patients with schizophrenia can be enhanced by aerobic exercise-induced BDNF upregulation.

**TRIAL REGISTRATION:**

The trial is registered at www.clinicaltrials.gov (NCT02864576)

